# Better Outcomes After Initiation of Disease-Modifying Therapy in Patients with Transthyretin Cardiac Amyloidosis

**DOI:** 10.3390/jcm15124546

**Published:** 2026-06-11

**Authors:** Makiko Nakamura, Teruhiko Imamura, Masaki Nakagaito, Ryuichi Ushijima, Koichiro Kinugawa

**Affiliations:** Second Department of Internal Medicine, University of Toyama, Toyama 930-0194, Japan; nakamuramk1979@gmail.com (M.N.);

**Keywords:** heart failure, tafamidis, ATTR-CM, troponin, biomarker

## Abstract

**Background**: Advances in diagnostic criteria for transthyretin cardiac amyloidosis (ATTR-CM) and expanded insurance coverage for bone scintigraphy have facilitated earlier detection of ATTR-CM. However, whether these changes have translated into improved clinical outcomes among patients receiving disease-modifying therapy remains uncertain, especially in non-high-volume centers. **Methods**: Consecutive patients with ATTR-CM who started disease-modifying therapy at our institute between May 2019 and March 2025 were retrospectively analyzed. Baseline characteristics and clinical outcomes were compared between the early period (2019–2021) and the late period (2021–2025). **Results**: A total of 31 patients (median age 77 years, 77% male) were included. Duration of heart failure was significantly shorter and the dose of loop diuretics at baseline was significantly lower in the late period (*p* < 0.05 for both). The prevalence of National Amyloid Center (NAC) stage I at baseline tended to be higher in the late period (75.0% versus 53.5%, *p* = 0.273). The cumulative incidence of worsening heart failure hospitalization and all-cause death was significantly lower in the late period (6.3% versus 44.2%, *p* = 0.024) during a median follow-up of 5 years. NAC stage I at baseline was independently associated with the lower primary outcome with an adjusted hazard ratio of 0.10 (95% confidence interval 0.01–0.90, *p* = 0.040). **Conclusions**: Patients with ATTR-CM in the late group experienced more favorable clinical outcomes after disease-modifying therapy, probably due to earlier diagnosis and therapeutic intervention, although further studies are warranted to verify the hypothesis.

## 1. Introduction

Systemic amyloidosis represents a diverse group of disorders caused by the deposition of misfolded proteins, arranged as amyloid fibrils within the extracellular space of multiple organs [1]. Transthyretin amyloidosis (ATTR) involves progressive deposition of fibrillar transthyretin (TTR) protein, whose physiological functions include carrying thyroid hormones and vitamin A. Transthyretin amyloidosis often affects elderly individuals (wild-type ATTR), likely because of an age-related decline in the homeostatic mechanisms that regulate protein metabolism. Transthyretin amyloidosis cardiomyopathy (ATTR-CM) progresses slowly and is often well tolerated clinically until significant wall thickening, severe diastolic dysfunction, atrial fibrillation, or conduction abnormalities occur [2]; however, in recent years, ATTR-CM has stood out as an emerging cause of aortic stenosis, unexplained left ventricular hypertrophy and heart failure (HF) with preserved ejection fraction, particularly in the elderly [3].

Historically, the diagnosis of ATTR-CM required a tissue biopsy. However, the introduction of a non-invasive diagnostic algorithm now enables ∼70% of cases to be diagnosed without histology [4]. In 2020, ^99m^Tc-pyrophosphate (PYP) scintigraphy received exceptional approval for reimbursement under the Japanese national health insurance system as a diagnostic method for ATTR-CM [5]. The Japanese Circulation Society also published guidelines on the diagnosis and treatment of cardiac amyloidosis in the same year [5], which may have theoretically facilitated early diagnosis for ATTR-CM nationwide, although yet to be demonstrated. Thanks to all the improvements in non-invasive diagnostic techniques, along with the development of efficacious therapies offering improvements in survival rates, ATTR-CM has been transformed from an incurable and infrequent condition to a relatively more diffuse and treatable disease, which physicians should take into consideration in the differential diagnostic processes in daily clinical practice [3].

In three randomized controlled trials evaluating disease-modifying therapies for ATTR-CM, it has been suggested that “less sick” patients [i.e., those with younger age, lower N-terminal pro-B-type natriuretic peptide (NT-proBNP), and lower New York Heart Association (NYHA) class] had better outcomes [6]. In the present study, we investigated whether the period of diagnosis (2019–2021 versus 2021–2025) was associated with clinical outcomes among patients with ATTR-CM in a non-high-volume single center in Japan, given recent advances in diagnostic strategies and healthcare system changes as stated above.

## 2. Methods

### 2.1. Patient Selection

We retrospectively included consecutive patients with ATTR-CM who started disease-modifying therapy between May 2019 and March 2025. All patients diagnosed with ATTR-CM who met the criteria of relevant clinical guidelines for the administration of disease-modifying therapy were initiated on such therapy. Day 0 was defined as the day of disease-modifying treatment [7].

In Japan, acoramidis and vutrisiran were approved for reimbursement for ATTR-CM in May 2025. Furthermore, eligibility for disease-modifying therapy was expanded to include patients diagnosed based on imaging criteria at the same time.

All patients were followed for five years after initiation of disease-modifying therapy or until May 2026, which was defined as the observation period for this study. The study was conducted in accordance with the Declaration of Helsinki. The use of anonymized clinical data was approved by the local institutional review board (IRB number, R2019166). Informed consent was obtained from all subjects involved in the study.

### 2.2. Clinical Management

All patients received standard medical therapy, including diuretics for chronic heart failure (HF), in addition to disease-modifying therapy. The management of HF was conducted by board-certified cardiologists.

Disease-modifying therapy was considered for discontinuation in several clinical scenarios after obtaining informed consent from patients and their relatives, including the need for non-pharmacological interventions, progression of frailty, cognitive decline, and progression of HF [8].

### 2.3. Baseline Characteristics

Medical records of all patients at the time of disease-modifying therapy initiation were retrospectively reviewed. Data on baseline demographic characteristics were collected, including the duration of HF, which was defined as the period from HF symptom onset until initiation of disease-modifying treatment, NYHA class, and the prevalence of atrial arrhythmia, catheter ablation for atrial arrhythmia, and pacemaker implantation.

### 2.4. Measurements of Biomarkers

All biomarkers were measured consistently throughout the study period using the same assay platforms. Plasma B-type natriuretic peptide (BNP), serum NT-proBNP, hemoglobin, serum albumin, and serum creatinine levels were measured immediately before disease-modifying therapy initiation as baseline data. Serum levels of cardiac troponin I and high-sensitivity cardiac troponin T (hs-cTnT) were measured within three months before disease-modifying therapy initiation. Plasma BNP and cardiac troponin I concentrations were measured using a commercially available assay (Abbott Japan, Matsudo, Japan). Serum NT-proBNP concentrations were measured by the Elecsys NT-proBNP immunoassay (Roche Diagnostics Ltd., Rotkreuz, Switzerland). hs-cTnT concentrations were measured using a commercially available assay (BML, Tokyo, Japan). Geriatric Nutritional Risk Index and National Amyloidosis Centre (NAC) ATTR stage, which is calculated based on NT-proBNP levels and estimated glomerular filtration rate, were also evaluated [9].

We divided all patients into two groups according to the period of disease-modifying therapy initiation: the early group (May 2019–January 2021) and the late group (February 2021–March 2025).

### 2.5. Measurements of Other Clinical Data

Electrocardiographic data, including QRS duration, and echocardiographic data, including interventricular septum thickness, left ventricular ejection fraction (LVEF), left ventricular mass index, the prevalence of aortic valve stenosis (moderate or severe), and tricuspid valve regurgitation (moderate or severe), were also collected. Left ventricular global longitudinal strain (GLS) had been assessed since 2021. The measurements of echocardiography were performed by physiological technicians and verified by board-certified cardiologists in accordance with the recommendations of the American Society of Echocardiography [10]. The interventricular septum thickness was measured using M-mode in the parasternal long-axis view. The LVEF was measured by the modified-Simpson method in the apical 4-chamber and 2-chamber views. The left ventricular mass index was calculated using the linear method with the Devereux and Reichek cube formula. Left ventricular GLS was assessed by speckle-tracking echocardiography. The type of disease-modifying therapy (tafamidis versus others) was also recorded. Concomitant medical therapy was also recorded. The dose of loop diuretics was expressed as the furosemide-equivalent dose.

### 2.6. Statistical Assessments

Statistics were performed using JMP Pro ver18.0 (SAS Institute Inc, Cary, NC, USA). Variables with *p* < 0.05 were considered significant. Continuous data were described as median and interquartile range and compared between two groups using the Mann–Whitney U test. Categorical data were compared between two groups by Fisher’s exact test.

All cohorts were divided into two groups: the early group and the late group. Patients were followed even after discontinuation of disease-modifying therapy. Cumulative incidence of composite outcome of worsening HF hospitalization and all-cause death was assessed by the Kaplan–Meier method and log-rank test as primary outcomes. HF hospitalization required symptoms and signs compatible with decompensated HF requiring inpatient medical treatment intensification, including the addition of non-pharmacological treatment under careful observation.

The impact of baseline disease severity on the cumulative incidence of the primary outcome was evaluated using univariable and multivariable Cox proportional hazards regression analyses. To avoid statistical overfitting, three variables, including NAC stage and concomitant medication with *p* < 0.05 in the univariable analyses, were included in the multivariable analyses as potential confounders.

## 3. Results

### 3.1. Baseline Demographics Data

Baseline demographic characteristics of the 31 enrolled patients are shown in Table 1, including 28 wild-type and 3 variant-type cases with the Val30Met mutation. The median age was 77 years, and 24 (77%) were male. The baseline NYHA classifications were as follows: 19 patients with class II, 11 patients with class III, and 1 patient with class IV. All patients underwent myocardial biopsy, and the amyloid type was confirmed by immunohistochemistry. Five (16%) patients had concomitant monoclonal gammopathy of undetermined significance (MGUS). Fourteen (45%) patients had atrial arrhythmia, and three of them had received catheter ablation. Nine (29%) patients had received cardiac pacemaker implantation.

**Figure 1:**
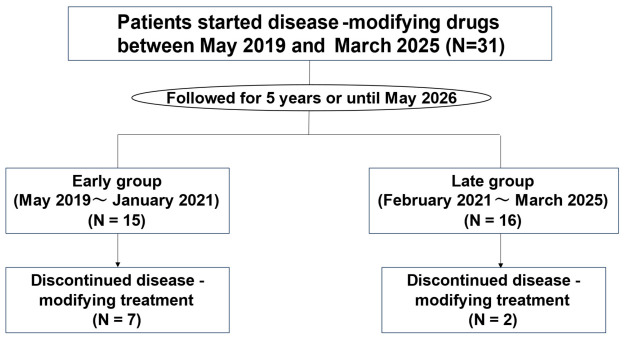
Patient flow in this study.

There were 15 patients in the early period (Figure 1). The median age, prevalence of wild-type ATTR, NYHA functional class, and prevalence of diagnosis by tissue biopsy, atrial arrhythmia, catheter ablation, and pacemaker implantation did not differ significantly between the early group and the late group. However, the duration of HF was significantly longer in the early group than in the late group (median 1.7 years versus 1.0 years, *p* = 0.020) (Table 1).

### 3.2. Baseline Laboratory Data

Hemoglobin levels were significantly lower in the early group, whereas serum albumin and creatinine levels did not significantly differ between the two groups (Table 2). The cardiac troponin I and hs-cTnT levels on median were 68 pg/mL and 0.046 ng/mL, respectively. Cardiac troponin I levels were significantly higher in the early group, and hs-cTnT levels were also higher in the early group. The median BNP and NT-proBNP levels were 211 and 1898 pg/mL, respectively. There were no significant differences in BNP and NT-proBNP levels at baseline, although those levels tended to be higher in the early group. There was no significant difference in NAC stage between the early group and late group (*p* = 0.277); however, the prevalence of NAC stage I tended to be higher in the late group (75.0% versus 53.3%, *p* = 0.273).

There were no significant differences in interventricular septal thickness, LVEF, and left ventricular mass index between the groups, although LVEF tended to be higher in the late group. Left ventricular GLS was measured only in the late group, with a median value of −10.0%. The prevalence of moderate or severe tricuspid regurgitation was numerically higher in the early group.

### 3.3. Disease-Modifying Therapy and Concomitant Medical Therapy

Overall, tafamidis therapy accounted for 93.6% (Table 3). One of the patients with variant-type was switched to vutrisiran after one year of tafamidis therapy for the indication of treatment in concomitant neuropathy. The remaining two patients with variant-type were treated with vutrisiran from the start. Nine patients discontinued tafamidis therapy until the end of the study period due to the progression of frailty, cognitive decline, HF severity, headache, colitis, and liver enzyme elevation; 7 of them (46.7%) were in the early group, and the remaining 2 (12.5%) were in the late group (Figure 1). As a result, disease-modifying therapy continued for a median of 20.5 (15.4, 52.7) months in the entire cohort. There was no significant difference in the duration of disease-modifying therapy between the early group and the late group (median 32.8 months versus 20.0 months, *p* = 0.457).

The prevalence of sodium-glucose cotransporter 2 (SGLT2) inhibitor use tended to be higher in the late group (81.3% versus 53.3%, *p* = 0.135), and the dose of loop diuretics was significantly lower in the late group (6 mg per day versus 20 mg per day on median, *p* = 0.008). There were no significant differences between the two groups in other medication variables (*p* > 0.05 for all).

### 3.4. Clinical Outcomes

**Figure 2:**
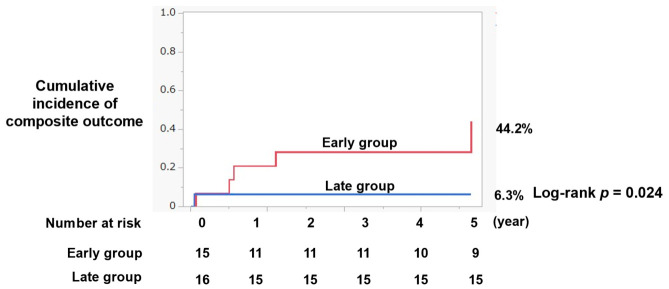
Comparison of the cumulative incidence of the composite outcome of worsening heart failure hospitalization and all-cause death between the early group and the late group.

There were 5 deaths and 6 worsening HF hospitalizations during the observational period. The cumulative incidence of the composite outcome of worsening HF hospitalization and all-cause death was significantly higher in the early group during a median follow-up of 5.0 (5.0, 5.0) years (44.2% versus 6.3%, *p* = 0.024; Figure 2). Event rates of the first worsening HF hospitalization were higher in the early group than in the late group (0.09 versus 0.03 events per year).

In the multivariable analyses, NAC stage I at baseline was independently associated with a lower risk of composite outcome, with an adjusted hazard ratio of 0.100 (95% confidence interval 0.010–0.898, *p* = 0.040; Table 4).

## 4. Discussion

In this study, we investigated the association between the period of disease-modifying therapy initiation and the clinical outcomes in patients with ATTR-CM. In the late group (since February 2021), the duration of HF was shorter, anemia was less progressed, the prevalence of NAC stage I was higher, and the dose of loop diuretics was lower. The cumulative incidence of the composite outcome of the worsening HF hospitalization and all-cause death was significantly lower in the late group. NAC stage I at baseline was independently associated with a lower risk of composite outcome.

### 4.1. Diagnosis of ATTR-CM in Clinical Practice

ATTR-CM can be diagnosed in the absence of histology in the setting of typical echocardiographic/cardiovascular magnetic resonance findings when ^99m^Tc-PYP, ^99m^Tc-3,3-diphosphono-1,2-propanodicarboxylic acid or ^99m^Tc-hydroxymethylene diphosphonate scintigraphy shows Grade 2 or 3 myocardial uptake of radiotracer and clonal dyscrasia is excluded by all the following tests: serum free light chain assay, serum, and urine protein electrophoresis with immunofixation [5]. Once ATTR-CM is confirmed, genetic testing should be performed to assess the presence of TTR mutations in order to differentiate between ATTR wild-type and ATTR variant type [11]. Although these non-invasive diagnostic capabilities have improved, access and interpretation remain inconsistent, particularly in community settings [12]. Subsequently, the Japanese Circulation Society released non-invasive diagnostic criteria in 2020, and ^99m^Tc-PYP scintigraphy was also reimbursed as a diagnostic method for ATTR-CM in the same year. ATTR-CM has been recognized as an underdiagnosed disease with a significant prevalence among HF patients with preserved LVEF [13,14]. This recognition formed the rationale for defining the late group as patients diagnosed from 2021 onward. The duration of HF at the time of disease-modifying therapy administration was significantly shorter in the late group, suggesting that clinicians became more aware of ATTR-CM and diagnosed it earlier than before.

### 4.2. Baseline Biomarker Level and Clinical Outcomes

In this study, baseline hemoglobin was higher, while troponin-I, hs-cTnT, and NT-proBNP levels were lower in the late group. The cumulative incidence of the composite outcome of worsening HF hospitalization and all-cause death was also lower in the late group. In the HELIOS-B trial, baseline NT-proBNP and troponin I levels were independently associated with risks of the composite outcome and all-cause mortality [15]. Another study also reported that increased hs-cTnT and BNP levels 1 year after tafamidis administration were independent predictors of higher cumulative risk of the composite outcome of all-cause death and HF hospitalization [16]. These biomarker levels may reflect the severity of myocardial damage in ATTR-CM and act as a surrogate marker for estimating prognosis following disease-modifying therapy, although further research is warranted to test this hypothesis.

### 4.3. Concomitant Medical Therapy and Clinical Outcomes

In the late group, the prevalence of SGLT2 inhibitor use tended to be higher, while the dose of loop diuretics was significantly lower than in the early group. Among patients with ATTR-CM, SGLT2 inhibitor therapy has been associated with better outcomes [17], and loop diuretic dose has been reported as an independent predictor of mortality [18,19]. The higher prevalence of SGLT2 inhibitor use, accompanied by dose reduction in loop diuretics, may have also contributed to the improved clinical outcomes in the late group. Therefore, we included the dose of loop diuretics in the multivariable analyses in this study. Further studies are warranted to establish optimal concomitant HF medical therapy in patients with ATTR-CM.

### 4.4. Changes in Baseline Disease Severity and Clinical Outcomes

In this study, patients in the late group had shorter HF duration, lower baseline cardiac troponin and natriuretic peptide levels, and a higher prevalence of NAC stage I. The baseline hs-cTnT and NT-proBNP levels have previously been reported to be useful biomarkers for evaluating the clinical outcomes following disease-modifying therapy administration [15,20,21]. Cox proportional hazards regression analyses showed that the NAC stage I at baseline was independently associated with a lower risk of composite outcomes. The NAC stage has been reported to be a universally applicable staging system that stratifies patients with both wild-type and variant-type ATTR-CM into prognostic categories [9], and our results were consistent with previous reports. The advances in the diagnosis of ATTR-CM and the expansion of insurance coverage for ^99m^Tc-PYP scintigraphy for ATTR-CM diagnosis might have led to earlier diagnosis and better outcomes since 2021 in our study.

In Japan, the prescription requirements for disease-modifying therapy were allowed for patients who were diagnosed by imaging-guided methods, in addition to those pathologically diagnosed with ATTR-CM, as of the end of May 2025. In this study, however, all patients were diagnosed with ATTR-CM by tissue biopsy, probably due to the study period. The prescription requirement change performed in May 2025 might also accelerate earlier administration of disease-modifying therapy. Evaluating the impact of the change in the prescription requirements on clinical outcomes is the next concern.

### 4.5. Study Limitations

This study was conducted retrospectively in a small sample size cohort at a single institute. We included several patients with variant-type ATTR-CM receiving medications other than tafamidis to evaluate temporal changes in baseline disease severity and clinical outcomes, rather than to evaluate the efficacy of tafamidis alone. There were several statistical differences in the medication prescription rates. We cannot completely exclude the prognostic impact of these medication updates between the two periods. We cannot exclude the influence of concomitantly performed non-pharmacological treatments. We cannot completely exclude the prognostic impact of potential confounders.

Each duration—one from symptom onset to ATTR-CM diagnosis, and the other from diagnosis to disease-modifying therapy initiation—was not evaluated. Serial changes in cardiac structure, functional status, and biomarkers, including ventricular wall thickness, LVEF, NYHA functional class, cardiac troponin and NT-proBNP levels, were not systematically available. There was no data on ^99m^Tc-PYP scintigraphy, exercise capacity, and quality of life. Left ventricular GLS data were only obtained in the late cohort. We did not measure comprehensive myocardial work indices, including global work index, global constructive work, global wasted work, and global work efficiency [22]. We could not have comprehensive cardiopulmonary exercise test data, which have a significant prognostic impact, given patients’ advanced frailty [23]. We could not assess ventricular-arterial coupling, expressed through systemic vascular resistance and myocardial work, which is another key parameter for assessing patients’ prognosis in this cohort [24]. Cardiac magnetic resonance is a key diagnostic and prognostic tool in ATTR-CM, but we did not collect comprehensive data regarding the modality. Instead, a tissue biopsy was performed in all participants.

## 5. Conclusions

Patients with ATTR-CM in the late group had lower baseline disease severity and more favorable clinical outcomes after disease-modifying therapy initiation. These findings might reflect earlier recognition of ATTR-CM and earlier therapeutic intervention in recent years, owing to the recent awareness of the disease and the innovation of non-invasive diagnostic modalities.

## Figures and Tables

**Table 1:** Comparison of Baseline characteristics.

	Total Cohort (*n* = 31)	Early Group (*n* = 15)	Late Group (*n* = 16)	*p* Value
Age (years)	77 (71, 80)	77 (71, 81)	78 (72, 80)	0.648
Sex, male *n*, (%)	24 (77.4%)	12 (80%)	12 (75%)	1.000
Wild-type *n*, (%)	28 (90.3%)	14 (93.3%)	14 (87.5%)	1.000
Body mass index (kg/m^2^)	22.8 (20.2, 24.9)	21.4 (20.4, 23.7)	24.1 (20.2, 26.0)	0.206
New York Heart Association functional class				0.398
Class I	0 (0%)	0 (0%)	0 (0%)	
Class II	19 (61.3%)	10 (66.7%)	9 (56.3%)	
Class III	11 (35.5%)	4 (26.7%)	7 (43.8%)	
Class IV	1 (3.2%)	1 (5.7%)	0 (0%)	
Duration of heart failure (years)	1.0 (0.5, 1.8)	1.7 (0.9, 3.7)	1.0 (0.8, 1.0)	0.020 *
Diagnosis by tissue biopsy	31 (100%)	15 (100%)	16 (100%)	NA
MGUS *n*, (%)	5 (16.3%)	2 (13.3%)	3 (18.8%)	1.000
Atrial arrhythmia *n*, (%)	14 (45.2%)	5 (33.3%)	9 (56.3%)	0.285
Catheter ablation for atrial arrhythmia *n*, (%)	3 (9.7%)	0 (0.0%)	3 (18.8%)	0.226
Cardiac pacemaker *n*, (%)	9 (29.0%)	5 (33.3%)	4 (25.0%)	0.704

MGUS, Monoclonal Gammopathy of Undetermined Significance; NA, not applicable.* *p* < 0.05 by Mann–Whitney U test or Fisher’s exact test as appropriate.

**Table 2:** Comparison of laboratory and echocardiographic data at the time of disease-modifying therapy initiation.

	Total Cohort (*n* = 31)	Early Group (*n* = 15)	Late Group (*n* = 16)	*p* Value
Hemoglobin (g/dL)	13.2 (12.1, 14.1)	12.3 (11.9, 13.2)	13.9 (13.4, 14.9)	0.002 *
Serum albumin (mg/dL)	4.0 (3.8, 4.2)	4.0 (3.8, 4.2)	4.0 (3.8, 4.2)	0.873
Serum creatinine (mg/dL)	1.1 (0.9, 1.2)	1.1 (0.9, 1.4)	1.0 (0.8, 1.2)	0.145
Troponin-I (pg/mL)	68 (49, 111)	102 (74, 152)	56 (41, 67)	0.002 *
High sensitivity-cardiac Troponin-T (ng/mL) (N = 28)	0.046 (0.032, 0.067)	0.060 (0.039, 0.078)	0.037 (0.032, 0.047)	0.050
Plasma B-type natriuretic peptide (pg/mL)	211 (103, 372)	247 (151, 506)	187 (87, 284)	0.105
Serum N-terminal pro-B-type natriuretic peptide (pg/mL)	1898 (1067, 3487)	2802 (1067, 5182)	1819 (1054, 2690)	0.324
Free light chain ratio (κ/λ)	1.37 (1.17, 1.65)	1.43 (1.27, 1.79)	1.30 (1.09, 1.30)	0.229
GNRI	122 (104, 142)	103 (96, 106)	104 (101, 104)	0.220
National Amyloidosis Center (NAC) stage				0.277
Stage I	20 (64.5%)	8 (53.3%)	12 (75.0%)	
Stage II	5 (16.1%)	4 (26.7%)	1 (6.3%)	
Stage III	6 (19.4%)	3 (20.0%)	3 (18.8%)	
NAC stage I *n*, (%)	20 (64.5%)	8 (53.3%)	12 (75.0%)	0.273
QRS duration (msec)	122 (104, 142)	111 (104, 149)	124 (100, 133)	0.607
Interventricular septum thickness (mm)	13 (12, 15)	14 (12, 15)	13 (13, 15)	0.747
Left ventricular ejection fraction (%)	57 (50, 59)	55 (40, 58)	57 (51, 65)	0.197
Left ventricular mass index (g/m^2^)	166 (133, 214)	177 (137, 220)	154 (130, 205)	0.363
Left ventricular GLS (%) (N = 15)	10.1 (8.5, 10.6)	NA	10.1 (8.5, 10.6)	NA
Aortic stenosis (>moderate)	2 (6.5%)	1 (6.7%)	1 (6.3%)	1.000
Tricuspid regurgitation (>moderate)	2 (6.5%)	2 (13.3%)	0 (0%)	0.226

GNRI, Geriatric Nutritional Risk Index; GLS, Global longitudinal strain; NA, not applicable. * *p* < 0.05 by Mann–Whitney U test or Fisher’s exact test as appropriate.

**Table 3:** Comparison of medical treatment at the time of disease-modifying treatment initiation.

	Total Cohort (*n* = 31)	Early Group (*n* = 15)	Late Group (*n* = 16)	*p* Value
Tafamidis therapy	29 (93.6%)	15 (100%)	14 (87.5%)	0.484
ACEI/ARB/ARNI	25 (80.7%)	11 (73.3%)	14 (87.5%)	0.394
Dose of beta-blocker (mg/day)	2.5 (0, 5)	1.25 (0, 10)	2.5 (0, 2.5)	0.804
Mineralocorticoid receptor antagonist	21 (67.7%)	9 (60.0%)	12 (75.0%)	0.458
Dose of loop diuretics (mg/day)	10 (0, 20)	20 (10, 40)	6 (0, 20)	0.008 *
SGLT2 inhibitors	21 (67.7%)	8 (53.3%)	13 (81.3%)	0.135
Dose of tolvaptan (mg/day)	0 (0, 3.75)	0 (0, 7.5)	0 (0, 0)	0.059

ACEI, Angiotensin-converting enzyme inhibitors; ARB, angiotensin II receptor blockers; ARNI, angiotensin receptor/neprilysin inhibitor; SGLT2, Sodium-glucose cotransporter 2. * *p* < 0.05 by Mann–Whitney U test or Fisher’s exact test as appropriate.

**Table 4:** Univariable and multivariable Cox proportional hazard ratio regression analyses for the composite outcome of heart failure hospitalization and all-cause mortality.

	Univariable Analyses		Multivariable Analyses	
	Hazard Ratio (95% CI)	*p*-Value	Hazard Ratio (95% CI)	*p*-Value
Age (years old)	1.018 (0.899–1.665)	0.783		
New York Heart Association functional class	2.335 (0.561–9.220)	0.220		
National Amyloid Center (NAC) stage I versus stage II/III	0.072 (0.009–0.605)	0.015	0.100 (0.011–0.898)	0.040
HF duration (years)	1.704 (1.002–2.831)	0.036	1.123 (0.647–1.951)	0.670
Hemoglobin (mg/dL)	0.796 (0.464, 1.373)	0.407		
Serum creatinine (mg/dL)	79.264 (4.971–1823.891)	0.003		
Serum albumin (mg/dL)	0.141 (0.011–2.329)	0.143		
Log_10_ BNP	15.574 (1.630–177.587)	0.019		
Log_10_ NT-proBNP	53.431 (3.397–1549.51)	0.009		
QRS duration (msec)	1.004 (1.010–1.068)	0.008		
LVEF (%)	0.937 (0.887–0.992)	0.020		
ACEI/ARB/ARNI	0.235 (0.052–1.054)	0.059		
Dose of beta-blockers (mg/day)	1.048 (0.897–1.171)	0.471		
Dose of loop diuretics (mg/day)	1.038 (1.002–1.072)	0.027	1.028 (0.983–1.072)	0.199
Mineralocorticoid receptor antagonist	1.405 (0.272–7.255)	0.684		
SGLT2 inhibitor	0.351 (0.078–1.573)	0.172		

NT-proBNP, N-terminal pro-B-type natriuretic peptide; ACEI, Angiotensin-converting enzyme inhibitors; ARB, angiotensin II receptor blockers; ARNI, angiotensin receptor/neprilysin inhibitor; SGLT2, Sodium-glucose cotransporter 2.

## Data Availability

Data are available upon reasonable request by the corresponding author.
